# Transmutation of long-lived fission products in an advanced nuclear energy system

**DOI:** 10.1038/s41598-022-06344-y

**Published:** 2022-02-09

**Authors:** X. Y. Sun, W. Luo, H. Y. Lan, Y. M. Song, Q. Y. Gao, Z. C. Zhu, J. G. Chen, X. Z. Cai

**Affiliations:** 1 School of Nuclear Science and Technology, University of South China, Hengyang, 421001 China; 2 Institute of Modern Physics, Chinese Academy of Sciences, Lanzhou, 730000 China; 3 Shanghai Institute of Applied Physics, Chinese Academy of Sciences, Shanghai, 201800 China

**Keywords:** Energy science and technology, Physics

## Abstract

Disposal of long-lived fission products (LLFPs) produced in reactors has been paid a lot attention for sustainable and clean nuclear energy. Although a few transmutation means have been proposed to address this issue, there are still scientific and/or engineering challenges to achieve efficient transmutation of LLFPs. In this study, we propose a novel concept of advanced nuclear energy system (ANES) for transmuting LLFPs efficiently without isotopic separation. The ANES comprises intense photoneutron source (PNS) and subcritical reactor, which consist of lead–bismuth (Pb-Bi) layer, beryllium (Be) layer, and fuel, LLFPs and shield assemblies. The PNS is produced by bombarding radioactive cesium and iodine target with a laser-Compton scattering (LCS) γ-ray beam. We investigate the effect of the ANES system layout on transmutation efficiency by Monte Carlo simulations. It is found that a proper combination of the Pb-Bi layer and the Be layer can increase the utilization efficiency of the PNS by a factor of ~ 10, which helps to decrease by almost the same factor the LCS γ-beam intensity required for driving the ANES. Supposing that the ANES operates over 20 years at a normal thermal power of 500 MWt, five LLFPs including ^99^Tc, ^129^I, ^107^Pd, ^137^Cs and ^79^Se could be transmuted by more than 30%. Their effective half-lives thus decrease drastically from ~ 10^6^ to less than 10^2^ years. It is suggested that this successful implementation of the ANES paves the avenue towards practical transmutation of LLFPs without isotopic separation.

## Introduction

Nuclear energy provides almost 10% of electricity production in the world^1^. Due to the low carbon release, nuclear energy plays an important role in facing the climate change. However, management of spent nuclear fuel (SNF) is becoming a major concern. After recovering U and Pu from SNF by PUREX process, most of the radioactive hazards leaving in high-level nuclear wastes are radiotoxic transuranics (TRUs) or long-lived fission products (LLFPs; ^79^Se, ^93^Zr, ^99^Tc, ^107^Pd, ^129^I, ^135^Cs, and ^137^Cs)^2–4^. While the TRUs inventory can be reduced significantly by recycling and incinerating them in advanced reactors, these LLFPs will likely dominate the long-term dose associated with radionuclide release from the geologic repository, owing to their high solubility in underground water and high activeness to move to the geosphere. To address this problem, transmutation of LLFPs into stable or short-lived isotopes has been suggested, which should follow the principle of “as low as reasonably achievable (ALARA)”^5,6^.

Nuclear transmutation relies mainly on either neutron capture reactions or photonuclear reactions^7–10^. Since transmutation of LLFPs is a particle consuming process, high particle flux or intensity is, in principle, needed. There are two key issues that affect transmutation efficiency of LLFPs: (1) transmutation cross sections; (2) isotopic compositions and density for sample target. Among these LLFPs, ^93^Zr and ^137^Cs can hardly be transmuted in a fast or a thermal neutron field since these radionuclides have small neutron capture cross sections^3,11,12^. The photonuclear transmutation becomes fascinating since it utilizes giant dipole resonance (GDR) reactions, which have slowly varied but medium GDR cross sections. After SNF partitioning, the selected seven LLFPs have mixed isotopic compositions^12,13^. Consequently, particle consumption on LLFPs transmutation would be much higher than the case when isotopic separation is adopted since other isotopes also capture or absorb particles. Among the LLFPs that need to be transmuted, ^79^Se, ^93^Zr, ^107^Pd, ^135^Cs, and ^137^Cs are not suitable for nuclear transmutation due to their relatively small isotopic abundances. To perform an efficient transmutation for interested LLFPs, isotopic separation is then required. However, no isotope-separation system for high-level nuclear wastes is so far technologically and economically feasible on an industrial scale^14^.

LLFPs transmutations in pressurized water reactors, fast spectrum reactors and accelerator-driven subcritical systems (ADS) have been studied to address the above-mentioned issues^15–17^. The feasibility of these reactors or systems depends on sufficient neutron excess per fission^18–21^. Even with isotopic separation, such transmutation on LLFPs needs at least 0.3 neutrons per fission. The ADS, in which high-flux neutrons are produced by spallation reactions with high-current proton accelerator, is designed particularly to produce energy and to transmute high-level nuclear wastes. The ADS availability has been evaluated^22–24^ and a preliminary ADS facility for studying nuclear transmutation is now being constructed in China^25,26^.

**Figure 1:**
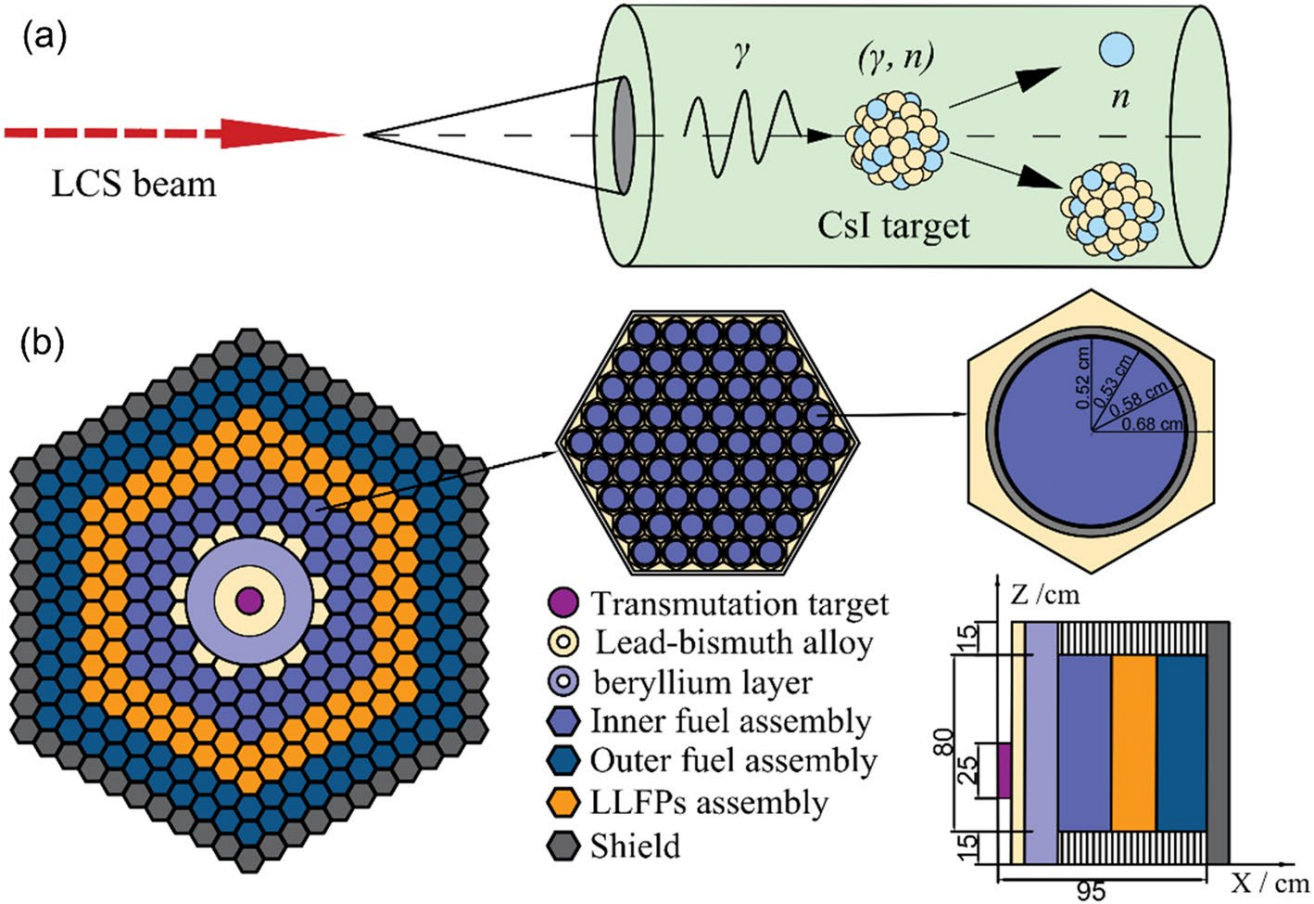
The concept of the ANES: (**a**) the generation of PNS and (**b**) the front view and side view of the ANES layout.

With the inspiration of ADS implementation, we introduce a novel concept on advanced nuclear energy system (ANES) that is driven by a photoneutron source (PNS) (see Fig. 1). The PNS is produced by bombarding a radioactive cesium and iodine (CsI) target with a laser-Compton scattering (LCS) γ-ray beam. As the LCS γ-ray beam has sufficient high intensity, such bombardment produces an intense PNS and meanwhile realizes photo-transmutation on radioactive cesium and iodine. The ANES is composed of the PNS and the reactor core which consists of lead–bismuth (Pb-Bi) layer, beryllium (Be) layer, and fuel, LLFPs and shield assemblies. In the ANES, both the transmutation and the energy production are accomplished. The fission energy production in the reactor core can be used to balance the energy consumption of PNS during transmutation, which means that the nuclear transmutation can be achieved without the need of external power supply. Moreover, one can expect to balance the initial cost for installing and operating the system, since this system could produce electricity and meanwhile lead to a large amount of heat generation during bombardment, making hydrogen fabrication possible^14^.

In this study, we present the conceptual design of the ANES for LLFPs transmutation without the requirement to isotopic separation. In our design, seven LLFPs are loaded in the reactor core for neutron transmutation and radioactive CsI target are handled with photo-transmutation while generating PNS. The implementation of the ANES is first introduced. Then the LCS energy spectrum and the ANES layout are optimized for improving the transmutation capability. In addition, the LCS beam intensity required for drive the ANES is evaluated. The results show that the proposed ANES concept may be a solution for transmuting LLFPs, albeit the existing LCS beam intensity is still a few orders of magnitudes lower than the requirement to drive the ANES at thermal power of 100 s MWt.

## Results and discussions

### ANES layout

The layout of the ANES is shown in Fig. 1. As an LCS γ-ray beam of high intensity irradiating the transmutation target (e.g., CsI target), a substantial population of neutrons are produced through photoneutron reactions, generating an intense PNS. Such PNS drives the ANES core, which is a subcritical one maintaining its intrinsic safety. The CsI target locates in the center of the ANES core. Considering a long-time irradiation, the cooling of the CsI target is achieved by circulation of liquid Pb-Bi alloy, which can endure higher energy density and longer operation cycle. A Be layer surrounding the Pb-Bi layer is adopted as neutron moderator. The combination of a 3 cm Pb-Bi layer and a 21 cm Be layer enhances the external neutron worth through neutron multiplication and moderation, as discussed later. The LLFPs without isotopic separation are loaded and transmuted with the excess neutrons leaked from the fuel assemblies. The $k_{{{\text{eff}}}}$*,* is designed to be ~ 0.98. The isotopic compositions of the LLFPs depend on the type of fuel, the neutron spectrum, and the irradiation history. The LLFPs used in this study are obtained from the burnup simulation of uranium dioxide fuel (see Methods).

The thermal power of ANES is designed to be 500 MWt. The ANES core, with a height of 110 cm and a diameter of 105 cm, contains 162 fuel assemblies, 78 LLFPs assemblies, and 60 shield assemblies. Each fuel assembly consists of 61 pins composed of uranium dioxide pellets covered by stainless steel cladding. Due to the intrinsic safety of the subcritical core, the ANES does not need control rods that are mandatorily used in a typical critical reactor. The LLFPs assemblies are arranged with two rows in the core, and the number of assemblies in inner and outer rows is 36 and 42, respectively. Like the fuel assembly, each LLFPs assembly incorporates 61 pins (43 LLFPs pins and 18 YD_2_ pins). The CsI target has a height of 25 cm and a radius of 3 cm. The shield assembly is made of stainless steel containing 6.48% natural B_4_C. In the burnup simulation of the ANES core, we use the dynamic refueling to keep a constant neutron flux over 20 years of operation. The detailed design parameters for the ANES are shown in Table 1.

**Table 1:** Design parameters of the ANES used in the simulation. The electric power is obtained supposing a thermal-electrical energy transfer efficiency of 40%.

Main parameters	Data used in this study
Type of fuel	UO_2_
Thermal power (MWt)	500
Electric power (MWe)	200
Core height (mm)	1100
Core diameter (mm)	1050
Number of fuel assemblies	60/102 (inner/outer)
Number of pins in each of fuel assembly	61
Pin diameter (mm)	5.8
Pellet diameter (mm)	5.2
^235^U enrichment (%)	23.3
Number of LLFPs assemblies	78
Number of pins in each of LLFPs assembly	61
Number of shield assemblies	60

### Production of PNS

**Figure 2:**
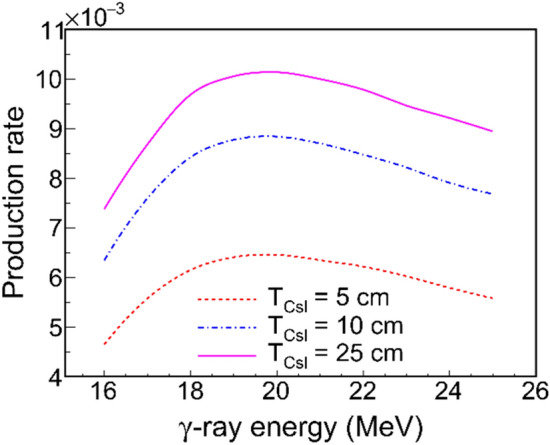
The $P_{n}$ (per γ photon) as a function of LCS γ-ray energy $E_{\gamma }^{max}$. Three kinds of CsI target thicknesses are used in the simulation while keeping the radius to be 3 cm.

For the PNS produced by an LCS γ-ray beam, the production rate $P_{n}$ is highly dependent on the γ-ray spectral distribution and the GDR cross section. When neglecting the nonlinear Compton scattering effect, the cut-off energy of the LCS γ-ray beam can be obtained with $E_{\gamma }^{max} = 4E_{L} \gamma^{2} /\left( {1 + 4E_{L} \gamma /m_{0} c^{2} } \right)$, where $E_{L}$ is the photon energy of the incident laser, $\gamma$ is the Lorentz factor of the electron beam from an advanced accelerator, and $m_{0} c^{2}$ presents the electron energy at rest. To maximize $P_{n}$, one can optimize $E_{\gamma }^{max}$ by varying the Lorentz factor for a fixed $E_{L}$. Figure 2 shows the dependence of $P_{n}$ on $E_{\gamma }^{max}$ for varying CsI target thicknesses, $T_{{{\text{CsI}}}}$. The $P_{n}$ increases first and then decrease with $E_{\gamma }^{max}$. Due to the convolution between the LCS γ-ray spectrum and the GDR cross section, the value of $P_{n}$ is peaked at $E_{\gamma }^{max}$ ~ 20 MeV. When $T_{{{\text{CsI}}}}$ is larger than 25 cm, the $P_{n}$ gets a saturation of 0.01, which is determined by the penetration depth of the LCS γ-ray beam. It is expected to produce γ-ray beam at an extremely high intensity of 10^17^ photon/s with the state-of-art of LCS facilities along with the advanced designs or concepts^27^. Consequently, the produced PNS could reach an intensity of 10^15^ photon/s.

### Performance of the ANES

**Figure 3:**
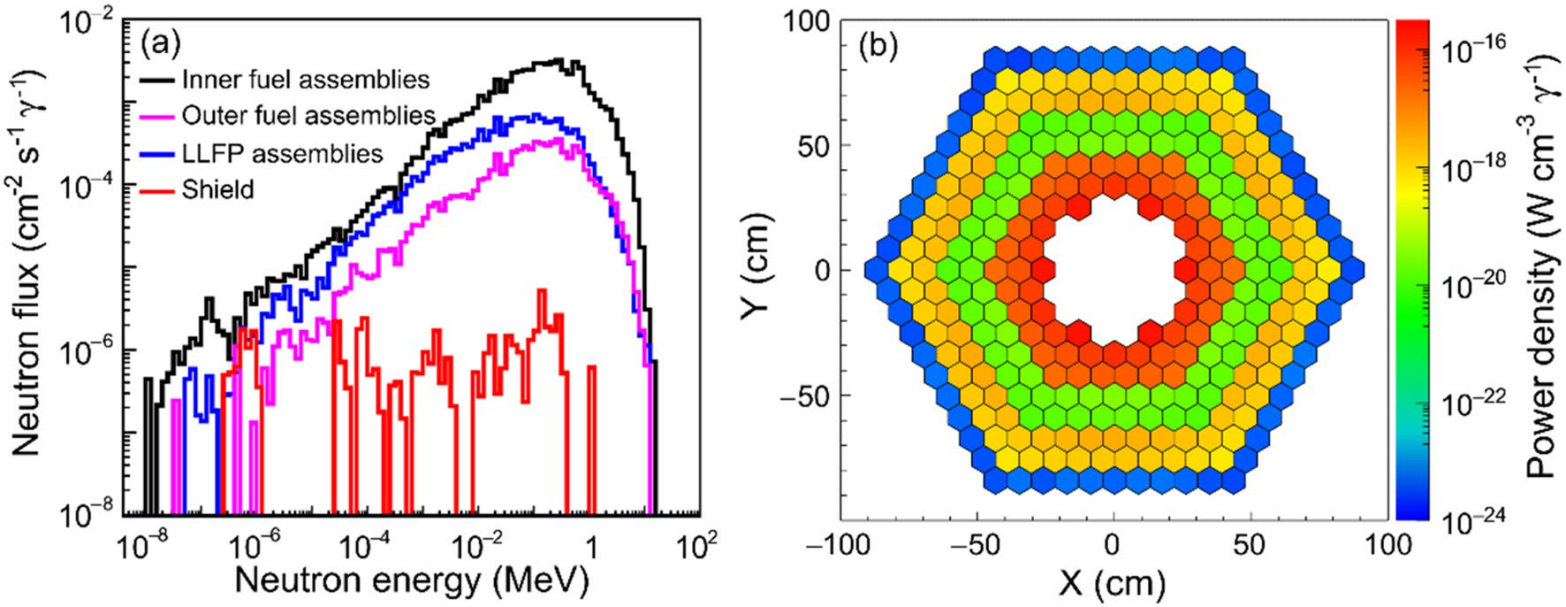
Neutron spectral pattern (**a**) and power density pattern (**b**) for the ANES.

Figure 3 shows the neutron spectrum and power density distributions in different assembly regions of the ANES core. In the region of LLFPs, the neutron spectrum is very similar to those in the region of fuel assemblies. The neutron flux decreases along the radial direction. In the shield region, the neutron flux is three orders of magnitudes lower than that in the inner fuel assemblies, indicating a good shielding for neutron radiation from the core. The power density varies mainly along with the neutron flux, as shown in Fig. 3b. The power density in the inner assemblies is obviously higher than that in the outer assemblies, which is in good agreement with the trend shown in Fig. 3a. A dip occurs in the region of LLFPs assemblies due to the absence of the fission process.

The performance of the ANES can be evaluated by a few key quantities including $k_{{{\text{eff}}}}$, $k_{{\text{s}}}$, neutron generation time (*Ʌ*) and effectively delayed neutron fraction ($\beta_{{{\text{eff}}}}$)^28,29^. The results for these quantities are displayed in Table 2. The initial value for $k_{{{\text{eff}}}}$ is 0.979. During two years of burnup, the $k_{{{\text{eff}}}}$ decreases slightly to 0.954. Accordingly, the sign of the system reactivity, $\rho$, is minus. According to Eq. (2), the $\varphi$ value is obtained to be 1.3, which is visibly higher than that given by the spallation neutron source^30^.

**Table 2:** Key parameters of the ANES in the initial moment.

Physical quantity	Value
Effective multiplication factor ($k_{{{\text{eff}}}}$)	0.979
Reactivity ($\rho$)	− 0.019
Effective multiplication factor for prompt neutrons ($k_{{\text{p}}}$)	0.977
Eigenvalue ($\alpha$)	− 0.003
Effective delayed neutron fraction ($\beta_{{{\text{eff}}}}$)	0.007
Neutron generation time (*Ʌ*) (μs)	0.523
Neutron worth of PNS ($\varphi$)	1.319
Sub-critical effective multiplication factor ($k_{{\text{s}}}$)	0.984

**Figure 4:**
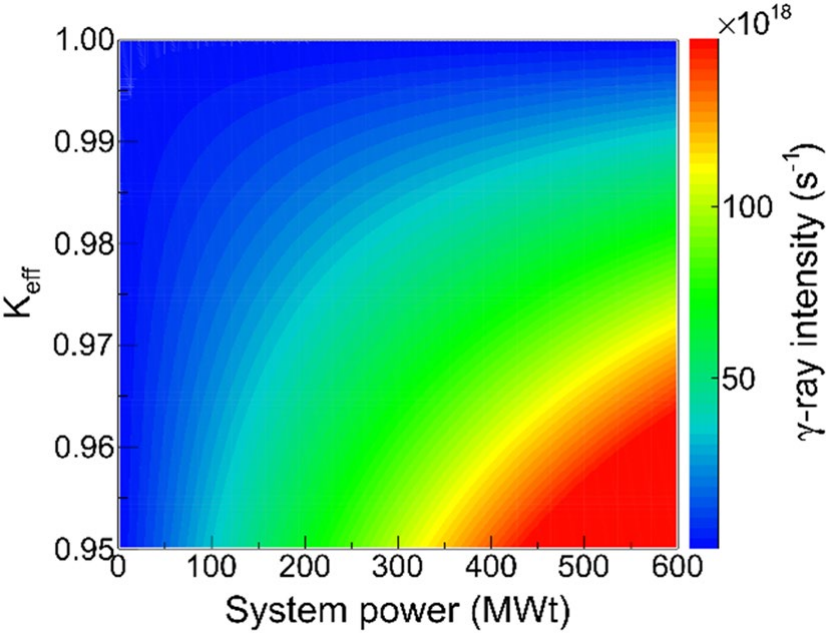
Dependence of the required $I_{\gamma }$ on thermal power $P_{t}$ and effective multiplication factor $k_{{{\text{eff}}}}$ considering the $P_{t}$ reaches the level of 100 MWt. An optimized value $P_{n} = 0.01$ is used for the calculation.

According to Eq. (4), the required $I_{\gamma }$ is dependent on both the $P_{t}$ and the $k_{{{\text{eff}}}}$. A contour plot for such dependence is shown in Fig. 4. It indicates that a higher $P_{t}$ requires a larger $I_{\gamma }$, which decreases with the increasing $k_{{{\text{eff}}}}$. To transmute the LLFPs efficiently, a thermal power of the order of 100 MWt is needed. Consequently, the $I_{\gamma }$ used to drive the ANES would exceed 10^19^ photons/s, which is almost two orders of magnitudes higher than that of existing LCS designs^12,27^. Recently, current and future LCS facilities used to generate MeV photon beams is reviewed and next generation photon sources based on advanced accelerator is outlooked^31^, which demonstrate a vivid future for developments of LCS facilities with ultra-high intensity. Here we continue to summarize a few novel concepts to enhance the $I_{\gamma }$, such as photon storage cavity^14,32^. This cavity aims to realize a high enhancement factor by increasing the stored laser power and reducing the laser size at focal point. It is expected that the cavity can achieve a 100 times improvement in γ-beam intensity. In addition, a conceptional design for a superconducting multi-turn energy-recovery linac (ERL) has been published, recently, by the international ERL community^33^. Such an ERL would produce a continuous-wave electron beam with extremely low emittance and very high current being capable of generating LCS photon beams with higher intensity.

In addition, the rather small cross section for LCS process (less than 665 mbarn) defines a physical restriction for the maximum photon flux which can be obtained from LCS facilities. A principle which overcomes this limitation has been discussed^31^. It uses a partially stripped ultra-relativistic ion beam, from which a resonant absorption of laser photons (which is in the Gbarn range) is followed by an atomic transition. Compared to the classical LCS process, unprecedented γ-beam intensity of the order of 10^17^ photons/s could be reached due to the massively larger cross section for the laser photon absorption. Combing these various concepts together, it would be possible to reach more than 10^19^ photons/s in the future.

### Transmutation of LLFPs

**Figure 5:**
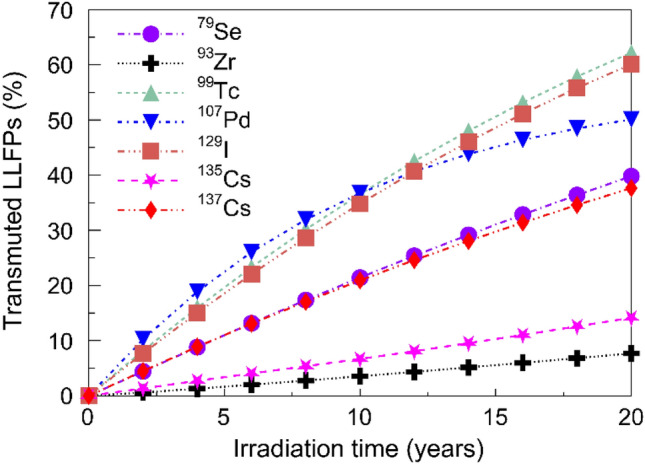
Transmutation of LLFPs over 20 years irradiation. The percentage of transmuted LLFPs after 20 years are in the order of ^99^Tc ≈ ^129^I > ^107^Pd > ^79^Se ≈ ^137^Cs > ^135^Cs > ^93^Zr.

The variation of transmuted LLFPs over 20 years of continuous irradiation was simulated. From Fig. 5 one can see that the mass of the transmuted LLFPs in the LLFPs assembly increases approximately linearly with the irradiation time. During 20-year irradiation, the transmutation percentages for ^79^Se, ^99^Tc, ^107^Pd, ^129^I and ^137^Cs are higher than 35%, whereas the reduction is less than 15% for both ^93^Zr and ^135^Cs. Similar results have been obtained in the fast neutron transmutation design^34^, although the transmutation of ^137^Cs is not considered therein. The low transmutation efficiency for ^93^Zr and ^135^Cs is mainly due to their relatively small capture cross sections.

The linear increase of transmuted LLFPs is further used to evaluate the effective half-lives $T_{{{\text{eff}}}}$, the TR and SR values for these LLFPs. The results are shown in Table 3, where the TR averaged over the irradiation time is considered. The $T_{{{\text{eff}}}}$ of the LLFPs decrease dramatically to the order of 10 years, while the radioactivity of LLFPs without transmutations can last more than 10^5^ years. For ^99^Tc, ^107^Pd, ^129^I and ^137^Cs, the TRs can achieve 2–3% per year. Although the TRs are relatively small, they are acceptable because the SRs > 1.0 would be more important for an ANES. In our study, the SRs are larger than 1.0 for ^79^Se, ^99^Tc, ^107^Pd, ^129^I and ^137^Cs, indicating the depletion of the LLFPs in the currently designed ANES. For ^93^Zr and ^135^Cs, the SRs are less than 1.0 due to small capture cross sections and large fission yields.

**Table 3:** Evaluated parameters obtained from SCALE output data of transmutation of LLFPs at 500 MWt.

LLFPs	$T_{{{\text{eff}}}}$ (year)	TR (%/year)	Transmutation in LLFPs assembly (g/year)	Production in fuel assembly (g/year)	SR
^79^Se	25.1	1.99	5.07 × 10^1^	4.40 × 10^1^	1.15
^93^Zr	131.6	0.38	8.66 × 10^2^	3.93 × 10^3^	0.22
^99^Tc	16.1	3.11	6.92 × 10^3^	3.83 × 10^3^	1.81
^107^Pd	20.0	2.50	6.65 × 10^2^	3.77 × 10^2^	1.76
^129^I	16.6	3.01	1.39 × 10^3^	8.56 × 10^2^	1.63
^135^Cs	70.4	0.71	2.39 × 10^3^	6.04 × 10^3^	0.39
^137^Cs	26.6	1.88	6.60 × 10^3^	6.17 × 10^3^	1.07

In the region of CsI target, hybrid transmutation (i.e., photo-transmutation and neutron transmutation) should be considered due to the mixed field of photons and neutrons. With the thermal power of 500 MWt, the transmutation capability for CsI target is shown in Table 4. The $T_{{{\text{eff}}}}$ for ^129^I, ^135^Cs and ^137^Cs decrease to less than 0.5 years according to Eq. (5), which includes the contribution of the photon and neutron transmutation. Compared with the only neutron transmutation on the LLFPs assemblies (see Table 3), the hybrid transmutation on CsI target can obtain two orders of magnitudes higher TR. In the photon field, the mass of transmuted ^129^I is a few times larger than those of transmuted ^135^Cs and ^137^Cs, which is mainly caused by the difference in isotope composition. In the neutron field, the transmuted ^129^I has a mass of 1.24 × 10^3^ g/year, which is comparable to that transmuted in the photon field (1.88 × 10^3^ g/year). However, in the ^135^Cs and ^137^Cs cases, the transmuted masses (induced mainly by neutron capture reactions on themselves) are less than the produced ones (induced mainly by their isotopes with mass number smaller than themselves). Since the cross section of ^134^Cs(n, γ) reaction is significantly higher than that of ^135^Cs(n, γ) reaction, and the isotope composition of ^134^Cs is increased with the irradiation time, the production of ^135^Cs can be larger than its consumption. The hybrid transmutation for ^129^I reaches 3.12 × 10^3^ g/year, which is almost one order of magnitude higher than those for ^135^Cs and ^137^Cs. This is mainly due to a high neutron capture cross section and a large isotope composition (see Table 5).

**Table 4:** Evaluated parameters obtained for transmutation of CsI target at 500 MWt. The tabulated data in photon field is obtained from Geant4 simulations, while the data in neutron field is obtained from SCALE simulations. The minus sign in neutron field on ^135^Cs (^137^Cs) suggests that the consumption of ^135^Cs (^137^Cs) is slower than its production.

LLFPs	$T_{{{\text{eff}}}}$ (year)	TR (%/year)	Transmutation in CsI target (g/year)		
			in photon field	in neutron field	in hybrid field
^129^I	0.2	254.67	1.88 × 10^3^	1.24 × 10^3^	3.12 × 10^3^
^135^Cs	0.4	123.85	3.85 × 10^2^	− 0.70 × 10^2^	3.15 × 10^2^
^137^Cs	0.3	151.51	9.25 × 10^2^	− 1.07 × 10^2^	8.18 × 10^2^

**Table 5:** Relative compositions of LLFPs in LLFPs assembly and their (n, γ) and (γ, n) parameters calculated by the TALYS software^45,46^.

Element	Isotope	Relative composition (wt%)	Natural half-life ($T_{1/2}$)	Neutrons capture cross section at 0.025 eV (barn)	(γ, n) parameters			
					*E*_th_ (MeV)	*Γ *(MeV)	*E*_max_ (MeV)	*σ*_max_ (mbarn)
Se	^76^Se	1.4 × 10^−4^	Stable	85.02	11.15	7.0	15.01	107
	^77^Se	0.032	Stable	41.33	7.52	5.2	17.04	141
	^78^Se	0.064	Stable	50.02	10.50	6.0	16.01	122
	^79^Se	0.096	3.27 × 10^5^ a	11.81	6.96	5.0	17.04	150
	^80^Se	0.190	Stable	0.59	9.91	5.0	16.01	128
	^82^Se	0.410	Stable	0.04	9.28	4.0	16.00	142
Zr	^90^Zr	0.190	Stable	0.01	11.97	4.5	17.00	192
	^91^Zr	6.790	Stable	1.30	7.19	4.5	17.00	182
	^92^Zr	8.060	Stable	0.23	8.63	3.2	16.01	159
	^93^Zr	8.590	1.53 × 10^6^ a	2.24	6.73	4.0	15.00	141
	^94^Zr	8.750	Stable	0.05	8.22	3.0	15.03	130
	^95^Zr	0.940	64.032 d	8.11	6.46	3.5	14.99	134
	^96^Zr	8.940	2.0 × 10^19^ a	0.02	7.85	4.5	14.98	103
Tc	^99^Tc	8.370	2.11 × 10^5^ a	22.80	8.97	3.7	15.99	202
Pd	^104^Pd	7.6 × 10^−5^	Stable	0.65	10.00	4.8	16.00	220
	^105^Pd	2.480	Stable	21.08	7.09	4.2	15.98	221
	^106^Pd	0.800	Stable	0.30	9.56	4.0	15.98	228
	^107^Pd	0.820	6.5 × 10^6^ a	9.53	6.54	4.0	15.98	232
	^108^Pd	0.380	Stable	8.57	9.22	4.1	15.98	194
	^109^Pd	0.120	13.701 h	24.20	6.15	3.8	16.01	208
I	^127^I	0.590	Stable	6.15	9.14	5.0	15.03	253
	^129^I	1.870	1.57 × 10^7^ a	30.29	8.99	5.0	15.56	300
Cs	^133^Cs	14.200	Stable	30.36	9.00	5.0	15.50	314
	^134^Cs	0.520	2.065 a	140.02	6.99	4.5	15.10	312
	^135^Cs	13.200	2.30 × 10^6^ a	8.41	8.78	4.5	15.00	316
	^136^Cs	0.012	13.16 d	13.36	6.83	4.0	15.02	322
	^137^Cs	13.500	30.08 a	0.27	8.30	3.5	15.00	325

It should be noted that the TR (averaged over the seven LLFPs) can reach 1.94% per year for the ANES (see Table 3). This is slightly higher than that (1.51% per year) in a fast reactor system^34^. Meanwhile, the ANES has additional advantage to transmute radioiodine and radiocesium by the hybrid transmutation.

### Optimal of the ANES layout

**Figure 6:**
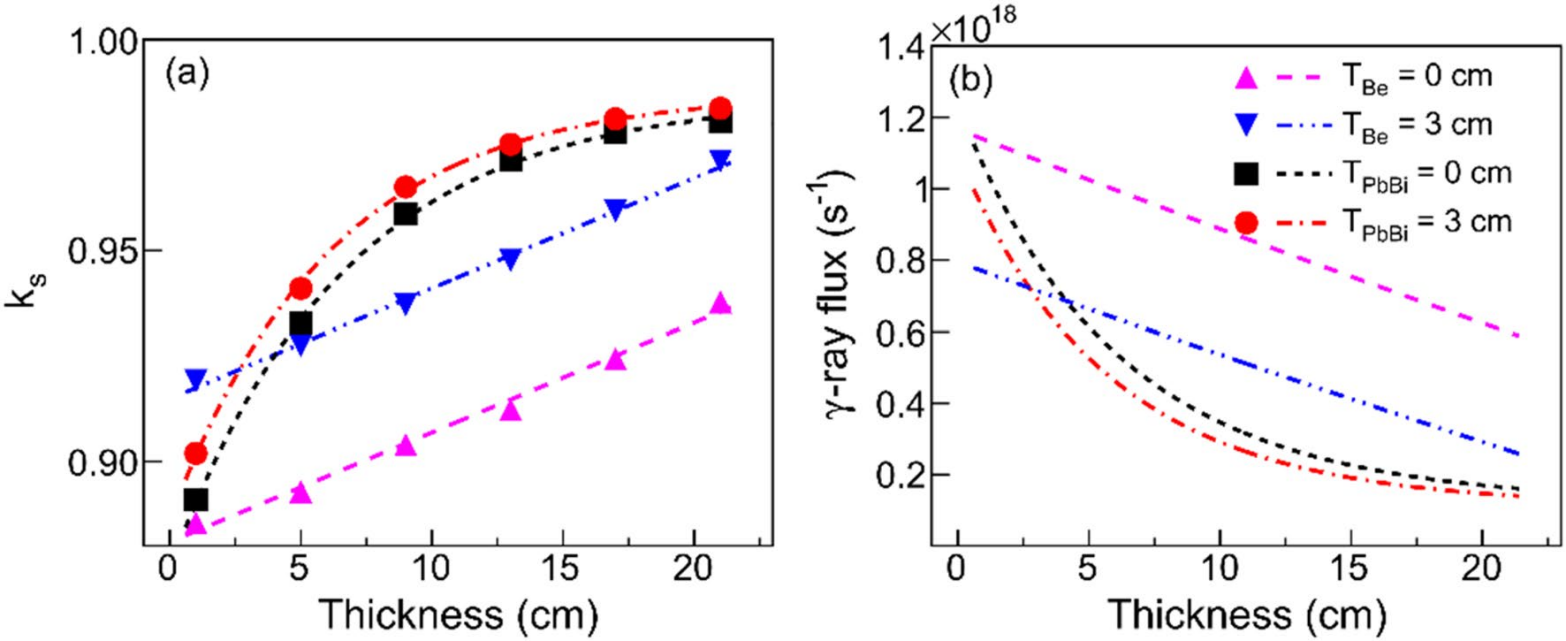
The dependence of $k_{{\text{s}}}$ on the thickness $T_{{\text{Be or PbBi}}}$ (**a**) and the γ-ray beam intensity required for maintaining a 1.0 MWt thermal power (**b**). The solid circle and long dashed-dotted line correspond to the simulated data and the fitting curve, respectively, for varying Be layer thickness but with a 3-cm-thick Pb-Bi layer. The solid square and short dashed line correspond to those obtained without the Pb-Bi layer. The inverse triangle and long dashed-dotted-dotted line correspond to the simulated data and the fitting result, respectively, for varying Pb-Bilayer thickness but with the 3-cm-thick Be layer. The regular triangle and long dashed line correspond to those without Be layer.

The neutron multiplier and moderator can be optimized in terms of absorption and moderation to ensure that the produced thermal neutrons can be effectively absorbed by the fuel assemblies in the core, which can thus enhance the neutron worth of PNS, $\varphi$. A Pb-Bi layer and a Be layer are used for neutron multiplication and moderation, respectively (see Fig. 1). The former also plays a key role in cooling the CsI target. The coolant and moderator dimensions are optimized to obtain a higher $k_{{\text{s}}}$. Figure 6a presents the dependence of $k_{{\text{s}}}$ on the thickness of either coolant or moderator, $T_{{\text{Be or PbBi}}}$ in units of cm. The fitting results are exponentially correlated functions and can be uniformly expressed as

1 $$ k_{{\text{s}}} = { }a_{1} + b_{1} \cdot {\text{exp}}(c_{1} T_{{\text{Be or PbBi}}} ), $$

where $a_{1}$, $b_{1}$ and $c_{1}$ are fitting parameters. In the absence of the coolant, $k_{{\text{s}}}$ is merely dependent on $T_{{{\text{Be}}}}$ with $a_{1}$ =  − 0.986, $b_{1}$ =  − 0.112 and $c_{1}$ =  − 0.150. As the coolant thickness is fixed to 3 cm, the value of $a_{1}$ remains unchanged, whereas *b*_1_ increases slightly to − 0.101 and $c_{1}$ decreases to − 0.162. It is shown a sub-linear trend because the stopping power of neutrons in the moderator increases with the thickness. When the moderator thickness is higher than 15 cm, the value of $k_{{\text{s}}}$ can approach 1.0.

In the absence of the moderator, $k_{{\text{s}}}$ is only dependent on $T_{{{\text{PbBi}}}}$ and we have $a_{1}$ = 1.076, $b_{1}$ =  − 0.200, and $c_{1}$ =  − 0.015. When considering a 3-cm-thick moderator, the value of $c_{1}$ is kept unchanged, whereas *a*_1_ increases slightly to 1.101 and $b_{1}$ increases to − 0.188. In this case, since the product of $c_{1}$ and $T_{{{\text{PbBi}}}}$ is much smaller than unity, Eq. (1) can be approximated as $k_{{\text{s}}} = { }a_{2} + b_{2} \cdot T_{{{\text{PbBi}}}}$ with $a_{2}$ and $b_{2}$ being the functions of $a_{1}$, $b_{1}$ and $c_{1}$. A quasi-linear trend is seen for the dependence of $k_{{\text{s}}}$ on $T_{{{\text{PbBi}}}}$, as shown in Fig. 6a. This trend is caused by the fact that the coolant can also result in (*n*, *xn*) reaction, which increases the neutron flux. As a result, the neutron multiplication does not attenuate with the coolant thickness.

According to Eq. (4), one can further obtain the correlation between $I_{\gamma }$ and $T_{{\text{Be or PbBi}}}$, as shown in Fig. 6b. Compared to the Pb-Bi layer, the Be layer has a more significant effect on both $k_{{\text{s}}}$ and $I_{\gamma }$. The $I_{\gamma }$ decreases rapidly with $T_{{{\text{Be}}}}$ and then gets saturated, whereas the $k_{{\text{s}}}$ has an opposite variation trend. When $T_{{{\text{Be}}}}$ is larger than 21 cm, the required $I_{\gamma }$ approaches 10^17^, which is approximately one order of magnitude lower than that before optimization (for example, in the absence of both Pb-Bi layer and Be layer).

**Figure 7:**
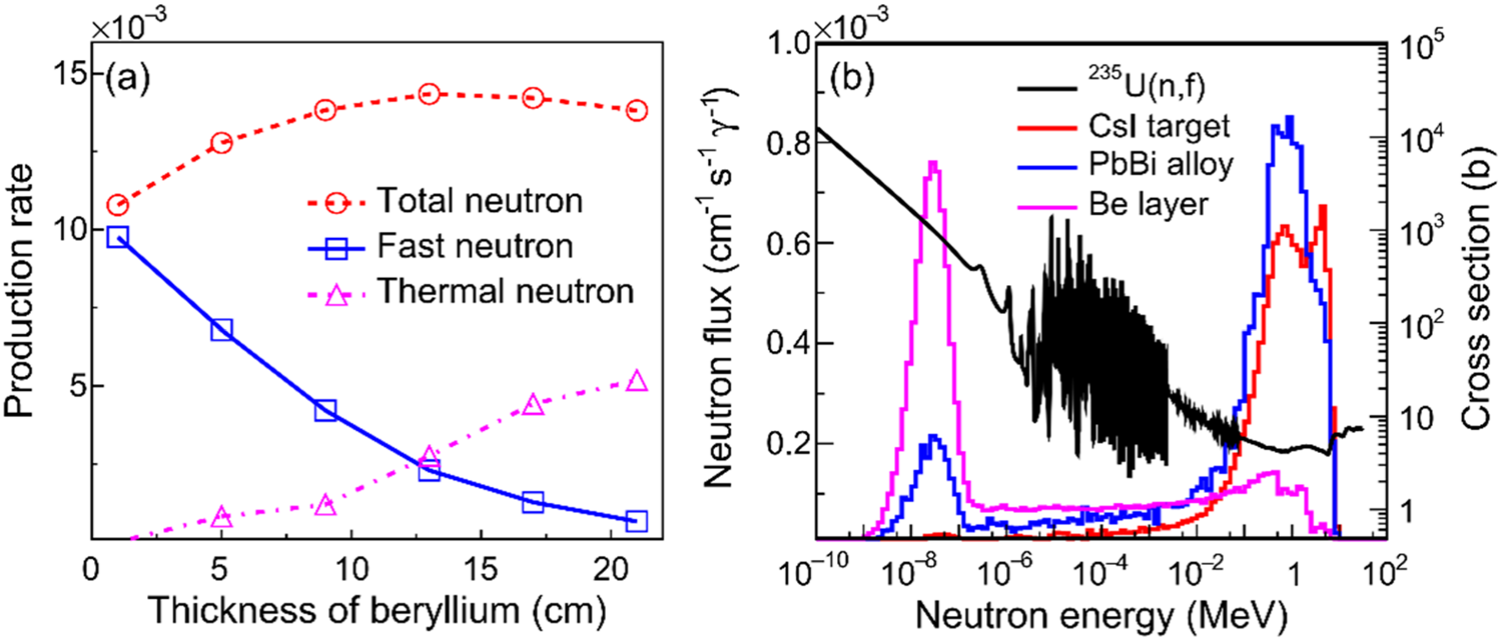
The $P_{n}$ (per γ photon) as a function of $T_{{{\text{Be}}}}$ with $T_{{{\text{PbBi}}}}$ = 3 cm (**a**) and spectral patterns of neutrons escaped from the CsI target (red solid line), the Pb-Bi layer with $T_{{{\text{PbBi}}}}$ = 3 cm (blue solid line) and the Be layer with $T_{{{\text{Be}}}}$ = 21 cm (magenta solid line) (**b**). The ^235^U (n, f) cross section (black sold line) is also shown.

The effects of beryllium thickness on neutron multiplication and on softening of neutron spectrum are obtained and shown in Fig. 7. When $T_{{{\text{Be}}}}$ increases, the number of fast neutrons declines and that of thermal neutrons rises. The $P_{n}$ reaches a maximum value for $T_{{{\text{Be}}}}$ = 13 cm and then decreases slightly due to the significant absorption of the neutrons produced therein. For $T_{{{\text{Be}}}}$ = 13 cm and $T_{{{\text{PbBi}}}}$ = 3 cm, the neutron multiplication is increased by a factor of to 0.3 compared to the case of $T_{{{\text{Be}}}}$ = 1 cm and $T_{{{\text{PbBi}}}}$ = 3 cm. After that, the major of fast neutrons can be moderated substantially to the thermal and epithermal region. For $T_{{{\text{Be}}}}$ = 21 cm and $T_{{{\text{PbBi}}}}$ = 3 cm, the spectra of neutrons emitting from the CsI target, Pb-Bi coolant and Be moderator are presented in Fig. 7b. The spectrum of photoneutrons has two peaks at around 1.0 MeV, which is probably induced by the neutrons from photonuclear (γ, n) and (γ, 2n) reactions at different energy regions. The neutron spectrum softens significantly in the moderator. As shown in Fig. 7b, these softened neutrons can enhance the fission cross sections of ^235^U by more than two orders of magnitudes, leading to a greater neutron worth $\varphi$ for the PNS.

**Figure 8:**
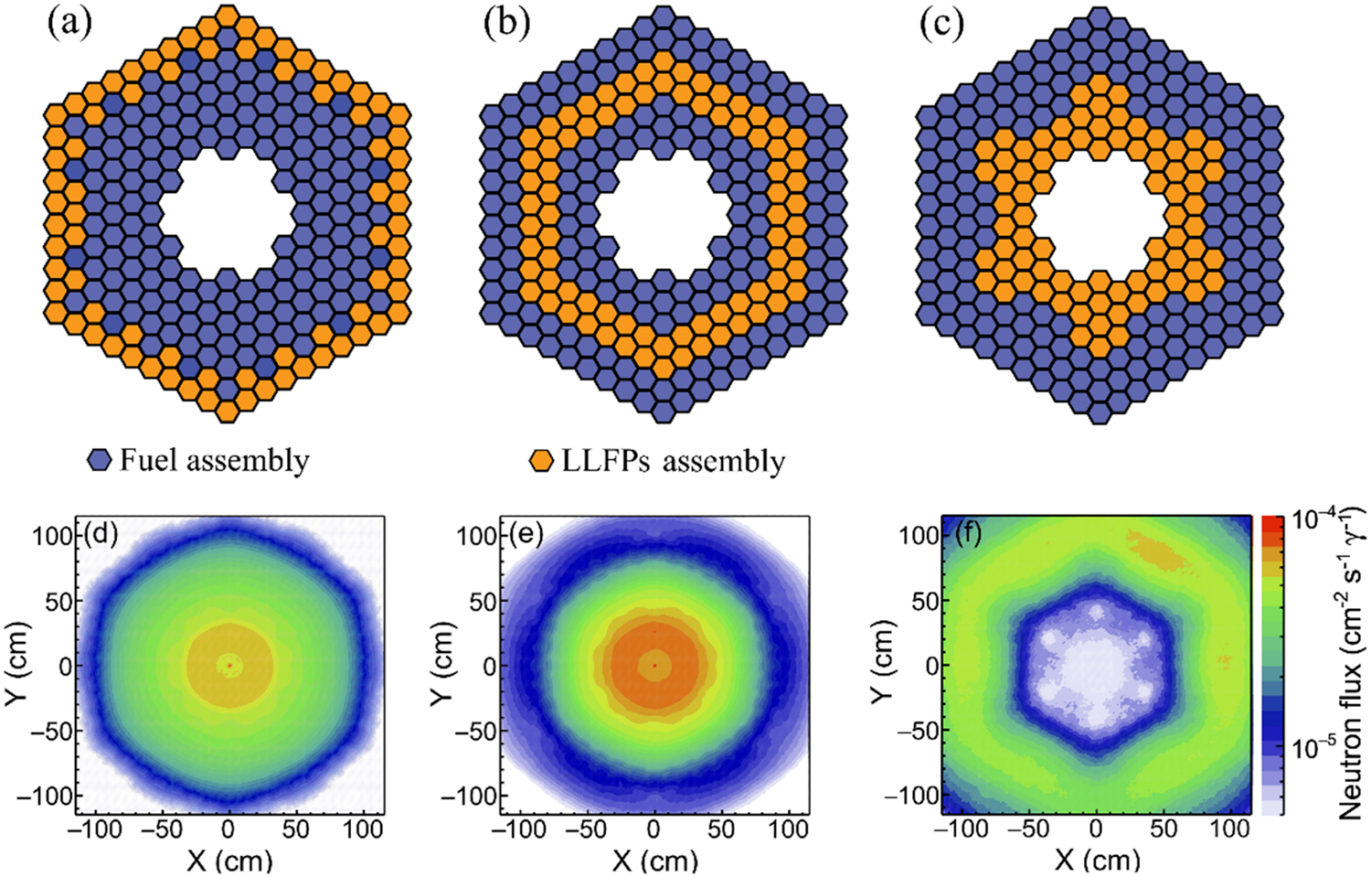
Three scenarios for LLFPs assembly in the outermost position (**a**), the middle position (**b**) and the innermost position (**c**) and their neutron spatial patterns presented for the LLFPs assembly in the outermost position (**d**), the middle position (**e**) and the innermost position (**f**). Only the neutrons from the fuel assembly are considered in the simulations. To keep the same $k_{{{\text{eff}}}}$ initially, the fuel enrichments are set to 17%, 23.3% and 24.1%, respectively.

Note that the LLFPs are neutron poisons in any transmutation system. For different arrangements of fuel and LLFPs assemblies, the resulting neutron spectra and fluxes could also vary, thus affecting the transmutation efficiency. To elucidate such effect, we consider three scenarios for arranging fuel and LLFPs assemblies. Scenario A, B and C show that the LLFPs assembly locates at the outermost, the middle and the innermost position of the ANES core, respectively. The detailed arrangements are shown in Figs. 8a–c. Note that scenario B corresponds to the layout shown in Fig. 1. In these scenarios, the neutron flux possesses different spatial patterns, as shown in Figs. 8d,e. Among these scenarios, scenario B shows the highest neutron flux in the region of LLFPs assembly. This is mainly caused by the convective effect of neutrons from the inner and outer fuel assemblies. In the scenario C, the LLFPs in the innermost position absorb many neutrons from the outer fuel assembly. As a result, the neutron flux shows a valley in the central zone, as shown in Fig. 8c.

**Figure 9:**
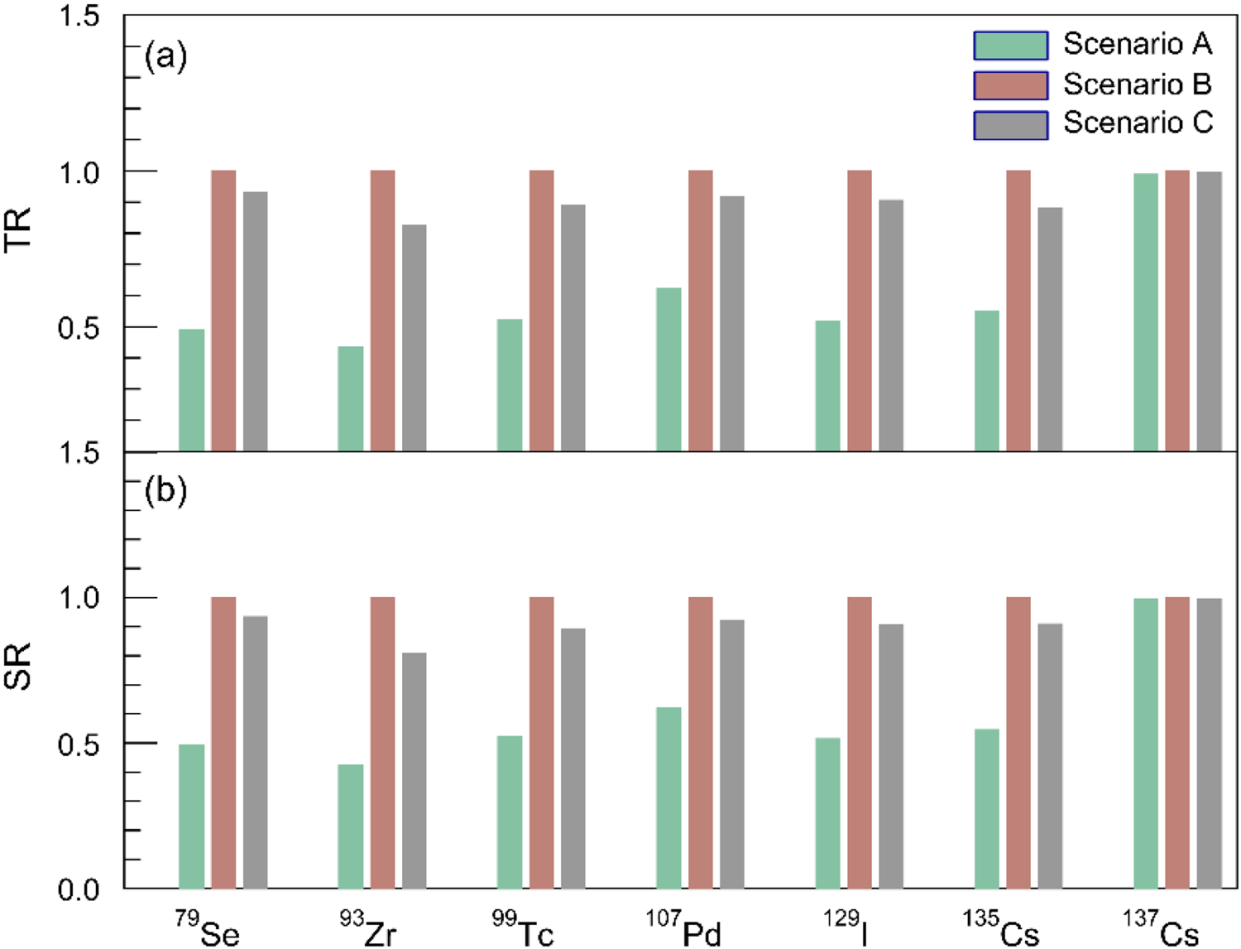
The TR (**a**) and SR (**b**) values for selected seven LLFPs. For comparison, the TR and SR values for scenario A and C are normalized by the ones for scenario B.

For the three scenarios shown in Fig. 8, the TR and SR values are further calculated. Figure 9 shows that scenario B results in the highest TR and SR for all LLFPs except for ^137^Cs. The transmutation on ^137^Cs is not sensitive to the scenarios due to its short half-life ($T_{1/2}$). The transmutation capability in scenario B is almost two times higher than in scenario A. As a result, we consider that scenario B would be the priority for LLFPs transmutation.

## Conclusion

We have presented a conceptual design of a ANES for efficient transmutation of LLFPs without isotopic separation. The ANES is driven by an intense PNS, which is produced by the energetic LCS γ-ray beam. The dimension of moderator and coolant is optimized, which enhances the $k_{{\text{s}}}$ and then decreases the required $I_{\gamma }$ by one order of magnitude. The performance of ANES and the transmutation capability are further analyzed. Especially, the $T_{{{\text{eff}}}}$, TR and SR values are predicted for LLFPs. Supposing the thermal power is 500 MWt and the irradiation time is 20 years, transmutation percentages are higher than 35% and the SRs are larger than 1.0 for ^79^Se, ^99^Tc, ^107^Pd, ^129^I and ^137^Cs. The $T_{{{\text{eff}}}}$ can thus be reduced from almost 10^6^ years to the level of 100 years, which dramatically decreases the cooling time of these LLFPs. Transmutation efficiency is also sensitive to the position of the LLFPs assembly. A proper arrangement for both the LLFPs assembly and the fuel assembly is found to realize an efficient transmutation. We conclude that the ANES driven by an intense PNS could be a good candidate for efficient transmutation on LLFPs without the need of isotopic separation.

## Methods

### Computational model and method

The production of LCS γ-ray beam and the following irradiation, which induces CsI transmutation and generates the PNS, were simulated with Geant4-MCLCSS and Geant4-GENBOD^35,36^. The transmutation cross sections required for simulation were taken from the ENDF-VII library^37^. The implementation of the ANES and its performance evaluation were performed with SCALE 6.1^38,39^. In the SCALE simulations, we taken the LCS spectral distribution as input and considered physical processes, including neutron-capture and photonuclear reactions. Furthermore, all burnup calculations were performed using either the TRITON t-depl or the STARBUCS sequence^39^, the $k_{{{\text{eff}}}}$ has a statistical error lower than 0.1%, and the reaction rate for evaluating the transmutation efficiency is within 0.5%.

### Selection of LLFPs

In general, the major LLFPs that need to be transmuted are ^79^Se, ^93^Zr, ^99^Tc, ^107^Pd, ^129^I, ^135^Cs and^137^Cs. These nuclides can cause long-term radioactivity during the geological disposal of SNF. We should note that the half-life of ^137^Cs is 30 years, which is much shorter than the other six fission products. Nevertheless, ^137^Cs is included in the transmutation inventory as an isotopic companion of ^135^Cs. This is because the latter can be transmuted effectively in the ANES without separation of isotopes. Namely, the transmutation of ^137^Cs can be regarded as a subsidiary of ^135^Cs. In addition, the CsI target used for the PNS plays an important role in the ANES system. The compositions of LLFPs were obtained from the burnup simulation of uranium dioxide pellets by fast breeder reactor core at 50 GWd/t for two years. The details of these compositions are presented in Table 5. Without isotopic separation, such compositions were used as the initial compositions of the LLFPs in the pins. All LLFPs were considered in metallic forms because their melting points are generally high, and the space volume for loading can be minimized^40^. Selenium is a metalloid element having a melting point of 221 °C and should be in a liquid phase at the operating temperature (expected to be about 600 °C) of the ANES. Therefore, ZnSe, which has a melting point of 1526 °C and a thermal conductivity of 19.04 W/(m∙K)^41,42^, was selected as a compound form that would be a solid phase when loaded into the system. Zirconium, Technetium and Palladium are transition metals with melting points of 1852 ℃, 2157 ℃ and 1554 ℃, respectively. Thus, we have chosen metallic forms for ^93^Zr, ^99^Tc and ^107^Pd, which could maintain a solid-state in the system. These metallic forms have thermal conductivities of 22.70, 50.60, and 71.80 W/(m∙K), respectively. Iodine is a halogen element having a melting point of 114 °C, and it is in a gas phase at the operating temperature of the system. BaI_2_ has a melting point of 711 °C and then was selected as a compound form that becomes a solid phase when loaded into the system. Cesium has a melting point of 28 °C. We chose Cs_2_CO_3_ as its chemical form which has a melting point of 610 °C and thermal conductivity of 2.88 W/(m∙K)^43^. These LLFPs are supposed to be dispersed homogeneously in the pins, which helps to transmute the LLFPs^44^.

### Selection of CsI target

The CsI target was selected for photo-transmutation due to the following considerations: ^129^I and ^135^Cs are problematic radionuclides since they have high radiotoxicity and long half-lives. The ^135^Cs strongly need isotopic separation for neutron-induced transmutation and the ^137^Cs is practically non-transmutable in any neutron field as aforementioned. Meanwhile, both the iodine and cesium elements have GDR cross sections as high as 300 mbarn, which is visibly higher than other elements (see Table 5) and may result in a significant transmutation. From the point of view of target fabrication, the two elements have the most stable chemical form in which three problematic radionuclides can be combined. The coolant temperature of the lead-based fast reactor ranges from 400 to 600 ℃, which is lower than the melting point of the CsI target (~ 620 ℃). In the simulations, the isotopic compositions for CsI target were employed according to SNF of a typical light water reactor^12,17^. These compositions are ^127^I (11.49%), ^129^I (38.51%), ^133^Cs (25.28%), ^134^Cs (0.01%), ^135^Cs (7.92%) and ^137^Cs (16.80%).

### Parameters of ANES

The neutron worth $\varphi$ represents the contribution of photoneutrons to the ANES core relative to fission neutrons^47^. As discussed above, $\varphi$ is an essential parameter for the system design and can be defined as

2 $$ \varphi = \frac{{1 - 1/k_{{{\text{eff}}}} }}{{1 - 1/k_{s} }}, $$

where $k_{{{\text{eff}}}}$ is the effective multiplication factor without considering the PNS, and $k_{s}$ is the multiplication factor considering PNS^48^. In our case, $k_{s}$ indicates the utilization efficiency of the ANES to the PNS. It is expressed by

3 $$ k_{s} = \frac{{\left\langle {F\Phi_{s} } \right\rangle }}{{\left\langle {F\Phi_{s} } \right\rangle + \left\langle S \right\rangle }} = \frac{{N - S_{0} }}{N} = 1 - \frac{{S_{0} }}{N}, $$

where $F$ is the creation operator, $S_{0}$ is the number of photoneutrons, $N$ is the total number of neutrons from nuclear fission and photonuclear process in the ANES, and $\Phi_{s}$ is the total neutron flux in the core. The thermal power of the ANES, $P_{t}$, is dependent on the averaged fission energy $E_{f}$, the LCS beam intensity $I_{\gamma }$, the production rate of photoneutrons $P_{n}$, and the average number of fission neutrons $\overline{\nu }$. In our study, it can be given by:

4 $$ P_{t} = E_{f} \cdot I_{\gamma } \cdot P_{n} \cdot \frac{{k_{{{\text{eff}}}} }}{{1 - k_{{{\text{eff}}}} }} \cdot \frac{1}{{\overline{\nu }}} \cdot \varphi . $$

It suggests that the $I_{\gamma }$ required for driving the ANES is inversely proportional to the $\varphi$ when keeping $P_{t}$ constant.

The $T_{{{\text{eff}}}}$ is defined as the effective half-life of radionuclides considering both transmutation process and natural decay in the core, which is crucial for evaluating the transmutation capability. Here $T_{{{\text{eff}}}}$ is expressed as

5 $$ T_{{{\text{eff}}}} = \frac{ln2}{{\lambda + \sigma \Phi_{s} }}, $$

where $\lambda$ and $\sigma$ are thenatural decay constant and the effective neutron capture cross section for transmuted radionuclides, respectively. Equation (5) can be approximated as $T_{{{\text{eff}}}} \approx \frac{ln2}{{\sigma {\Phi }_{s} }}$ as the $\lambda$ are extremely smaller than the product of $\sigma$ and $\Phi_{s}$, indicating that an efficient transmutation can reduce significantly the $T_{{{\text{eff}}}}$.

For a transmutation system, transmutation rate ($TR$) and support ratio ($SR$) are also two important parameters^34^. Here, $TR$ is the ratio of the amount of transmuted LLFPs to those initially loaded in a transmutation system, and $SR$ is the ratio of the amount of transmuted LLFPs to that of produced ones. The expressions of $TR$ and $SR$ are

6 $$ TR = \frac{N\left( 0 \right) - N\left( t \right)}{{tN\left( 0 \right)}}, $$

7 $$ SR = \frac{N\left( 0 \right) - N\left( t \right)}{{YMt}}. $$

Here $$N\left( 0 \right)$$ and $$t$$ are the total initial atomic number of LLFPs and irradiation time, respectively; $$Y$$ and $$M$$ are the LLFPs yield per fission of fuel materials and the total fission rate in the ANES core. When the value of $$\sigma \Phi_{s} t$$ is small enough, the $$TR$$ and $$SR$$ can be simplified as $$\sigma \Phi_{s}$$ and $$N\left( 0 \right)\sigma \Phi_{s} /YM$$, respectively. If $$SR$$ > 1.0, those self-produced LLFPs could be transmuted during the operation of the ANES. In our study, a direct approach to enhancing $$SR$$ is to increase the number of initially loaded LLFPs. However, the $$TR$$ will be decreased due to the neutron self-shielding effect in the loaded zone. As a result, it is imperative to balance the $$TR$$ and $$ SR$$ for LLFPs of great interest.
